# Technical Report: A Comprehensive Comparison between Different Quantification Versions of Nightingale Health’s ^1^H-NMR Metabolomics Platform

**DOI:** 10.3390/metabo13121181

**Published:** 2023-11-30

**Authors:** Daniele Bizzarri, Marcel J. T. Reinders, Marian Beekman, P. Eline Slagboom, Erik B. van den Akker, on behalf of the BbmriNl

**Affiliations:** 1 Molecular Epidemiology, Department of Biomedical Data Science, Leiden University Medical Center, 2333 ZC Leiden, The Netherlands; 2 Leiden Computational Biology Center, Department of Biomedical Data Science, Leiden University Medical Center, 2333 ZC Leiden, The Netherlands; 3 Delft Bioinformatics Lab., Department of Intelligent Systems, TU Delft, 2628 XE Delft, The Netherlands; 4 Max Planck Institute for the Biology of Ageing, 50931 Cologne, Germany

**Keywords:** NMR metabolomics, epidemiology, re-quantification, multivariate risk models, nightingale health

## Abstract

^1^H-NMR metabolomics data is increasingly used to track health and disease. Nightingale Health, a major supplier of ^1^H-NMR metabolomics, has recently updated the quantification strategy to further align with clinical standards. Such updates, however, might influence backward replicability, particularly affecting studies with repeated measures. Using data from BBMRI-NL consortium (~28,000 samples from 28 cohorts), we compared Nightingale data, originally released in 2014 and 2016, with a re-quantified version released in 2020, of which both versions were based on the same NMR spectra. Apart from two discontinued and twenty-three new analytes, we generally observe a high concordance between quantification versions with 73 out of 222 (33%) analytes showing a mean ρ > 0.9 across all cohorts. Conversely, five analytes consistently showed lower Spearman’s correlations (ρ < 0.7) between versions, namely acetoacetate, LDL-L, saturated fatty acids, S-HDL-C, and sphingomyelins. Furthermore, previously trained multi-analyte scores, such as *MetaboAge* or *MetaboHealth*, might be particularly sensitive to platform changes. Whereas *MetaboHealth* replicated well, the *MetaboAge* score had to be retrained due to use of discontinued analytes. Notably, both scores in the re-quantified data recapitulated mortality associations observed previously. Concluding, we urge caution in utilizing different platform versions to avoid mixing analytes, having different units, or simply being discontinued.

## 1. Introduction

Targeted 1H-NMR Metabolomics has rapidly gained popularity as a cost-effective and comprehensive method to perform metabolic profiling and risk prediction in large epidemiological studies. Various of such metabolomics-based age predictors were constructed; for example *MetaboAge*, an indicator of several future cardiovascular diseases [1] and *MetaboHealth* that predicts multiple health conditions and all-cause mortality [2]. Thus far, targeted 1H-NMR Metabolomics has shown promise to predict COVID hospitalization [3], various disease outcomes [4,5,6], and a plethora of conventional clinical risk variables [7].

Targeted 1H-NMR approaches focus on the analysis of a limited and pre-defined set of analytes, whose associated peaks consistently appear at relatively fixed positions in the overall NMR spectrum of a specific biomaterial and can therefore be robustly quantified [8]. Each of the associated peaks are quantified according to standardized rules and then transformed into absolute quantities with the aid of reference compounds [8]. While each change in the assayed biomaterials or isolation protocols would necessitate a considerable effort to re-calibrate a ^1^H-NMR-based quantification setup, a rigid standardization of both the input material and the laboratory routines would allow for a cost-effective and metabolome profiling on an epidemiological scale [9,10]. 

Nightingale Health Plc is a major commercial supplier of targeted 1H-NMR metabolomics data with bench-to-data solutions for human serum, plasma, or urine, for a limited number of metabolic markers. Large consortia like BBMRI.NL [1], FINSK/THL [5], COMETS [11], and, more recently, UK-Biobank [3] have set out to enrich their population studies with 1H-NMR metabolomics profiling and to date have accumulated data in respectively ~35,000, ~40,000, ~46,000, and ~300,000 samples. Sample handling and processing inevitably varies during and between such large efforts and may introduce variation in the data that could potentially impede replication efforts. In parallel with their metabolomics profiling efforts in UK-Biobank, Nightingale Health updated the way their analytes are quantified to further improve the calibration of 37 of their analytes with clinically measured counterparts. While such updates constitute a further optimization of this biomarker platform, it may also introduce systematic changes with respect to previously assayed or longitudinal studies [12,13,14,15]. 

Here, we set out to quantify to which extent the most recent updates of the quantification procedure by Nightingale affected the reported analytes, and to what extent this could influence replication of previous findings. To this end, we analyzed the Spearman’s correlations (ρ) of ~220 metabolic analytes quantified by Nightingale Health across three different platform versions (2014, 2016, and 2020) leveraging samples for which multiple quantifications were performed on basis of the identical NMR spectra. We found that, while many analytes present a high degree of Spearman’s correlation between versions, a number of analytes present a moderate to low Spearman’s correlation. In addition, we demonstrate that the effect on multi-analyte scores may differ, and thus ideally would require their renewed validation for each platform update. For example, the *MetaboHealth* score exhibits similar associations with time to death, whereas the metabolomics-based age predictor (*MetaboAge*) could no longer be readily applied due to use of discontinued metabolites yet could be successfully retrained on the new platform version and showed similar associations with disease outcomes. 

## 2. Materials and Methods

### 2.1. Dataset Descriptions

The Dutch Biobanking and BioMolecular resources and Research Infrastructure (BBMRI.NL) is a large consortium composed of 28 Dutch cohorts, which quantified their samples with the Nightingale Health platform in different time points, allowing an investigation on the platform differences over the years. About 25,000 samples from 26 cohorts were quantified during the first wave in 2014. A second wave of 10,000 samples was then obtained in 2016, including some longitudinal time-points and 2 new cohorts. Finally, after the 2020 update of the platform, the entire BBMRI.NL (35,000 samples from 28 cohorts) was re-quantified to have comparable measures to other Consortia. 

#### 2.1.1. BBMRI.NL

BBMRI.NL (https://www.bbmri.nl/, last access: 1 October 2023) is a Dutch Consortium which includes a total of 35,000 samples from the following 28 Dutch biobanks: ALPHAOMEGA [16], BIOMARCS [17], CHARM [18], CHECK [19], CODAM [20], CSF [18], DMS [21], DZS_WF [22], ERF [23], FUNCTGENOMICS [24], GARP [25], HELIUS [26], HOF [27], LIFELINES [28], LLS_PARTOFFS [29], LLS_SIBS [29], MRS [18], NESDA [30], PROSPER [31], RAAK [32,33], RS [34], STABILITEIT [35], STEMI_GIPS-III [36], TACTICS [32,33], TOMAAT [32,33], UCORBIO [37], VUMC_ADC [35], VUNTR [38]. Complete descriptions and ethics statement of each cohort is added to the Supplementary Materials.

Metabolomics Dataset: Nightingale Health performed the quantification of high throughput proton Nuclear Magnetic Resonance (^1^H-NMR) for the EDTA plasma for BBMRI.NL in separate waves (Table 1). The first wave was performed in 2014, on a great portion of the data (~25,000 samples). The second wave was performed in 2016 to quantify ^1^H-NMR metabolomics in the cohorts HOF and STABILITEIT, but also to quantify follow-ups sampling from different cohorts. Finally, in 2021 a re-quantification was performed to the entire dataset to update the metabolomics measurements to the latest platform version (platform version 2020).

#### 2.1.2. The Leiden Longevity Study

The Leiden Longevity Study is one of the cohort included in BBMRI.NL, which comprises a first generation subgroup of long-lived parents (LLS-SIBS, age = 89 ÷ 103 years old) and a second generation which includes their middle-aged offspring with the relative partner (LLS-PAROFFS age median = 30 ÷ 79 years old) [29]. 

Metabolomics Dataset: While only one sample collection was performed on the older individuals of LLS-SIBS [998 individuals], there are three time-points available for LLS-PAROFFS drawn with ~3 years gap one after the other (IOP1, IOP2 and IOP3) (Table 1). The first-time point (IOP1, 2313 individuals) was quantified during the first wave in 2014, while the second and third samples measurements (IOP2 and IOP3, respectively, 670 and 498 individuals) were included in the second wave, with the platform version 2016. All the samples were then re-quantified in 2021 with the rest of BBMRI.NL data (Table 2). The last column of the Table 2 shows the number of common samples after the quality control of the two datasets, described in the next paragraph.

### 2.2. Comparison of the Metabolomic Analytes

Preprocessing: All the three versions of the metabolomics assays were run by Nightingale Health on EDTA-plasma samples handled by the BBMRI.NL cohorts. More than 220 analytes are included in all nightingale platform; however, we decided to mostly focus our attention on the 63 mutually independent analytes used to build the previous metabolomics-based models [1,2,7]. However, since 2 of these analytes were discontinued (hdl2_c and hdl3_c), we substituted them with 4 biologically equivalent analytes, upon Nightingale’s Health advice (xl_hdl_c, l_hdl_c, m_hdl_c, s_hdl_c) (lists in Supplementary Materials), which are available in all datasets. We then removed samples with more than 1 missing value, more than one zero and more than one outlier, defined as having a concentration more than 5 standard deviations away from the mean of the analyte.

Analyses: We used Spearman’s correlation (ρ) to measure the strength and direction of monotonic associations between the analytes in the different versions of the platform. We also used a median absolute distance to evaluate the error of Nightingale Health’s analytes to the clinically measured values. The median absolute distance is obtained by using median and standard deviations of the clinical measures to scale all measures (both clinical and Nightingale quantifications) to have comparable results.

### 2.3. MetaboHealth Score

Preprocessing: The *MetaboHealth* score was applied to both the datasets (the first wave and the re-quantified), according to the description by Deelen et al. [2], using the R-package MiMIR [39]. First, a logarithm transformation was applied to the analytes, while adding a value of 1 to all analytes containing any zero. A z-scale normalization was then applied to the log-transformed analytes in each cohort separately. Finally, the coefficients as indicated by Deelen et al. [2] were applied to the dataset.

Analyses: Once we obtained the score, we used Spearman’s correlation to compare the differences in *MetaboHealth* score before and after re-quantification. Cox proportional hazard models are then used to test the associations between the two *MetaboHealth* scores and time to death.

### 2.4. MetaboAge

Preprocessing: The quality control process used for the dataset in the first wave of measures (data 2014) is discussed in details in our previous publications [1,7]. We used the same steps also in the re-quantified dataset. From the above-mentioned list of 65 analytes, we decided not to consider analytes with low detection rates in several cohorts (citrate and 3-hydroxybutyrate). We then excluded cohorts with several problems in the 65 selected analytes. VUNTR ( 3559 samples) has high levels of missingness in pyruvate and glutamine, while CODAM (145 samples) presented outliers in several metabolic features [20,38]. We also removed samples with 1 or more missing value (65 samples), one or more zeroes per sample (1 sample), and one or more concentration more than 5 times the standard deviations away from the general mean of the feature (644 samples). The remaining 265 missing values (0.021% of the remaining values) were imputed using nipals (in the R package pcaMethods). The final dataset, comprising 20,366 samples and 63 analytes, was z-scaled to have comparable concentrations across all features.

Analyses: Due to discontinued analytes, we had to retrain the models and we decided to train 2 different types of models: a linear regression model, to maintain the model as close as possible to the previous version, and an ElasticNET regression, which avoids overfitting thanks to a regularization technique. To train and evaluate both models we employed a 5-Fold Cross Validation scheme. During the training of the ElasticNET model we fixed the mixing parameter α to 0.5 and optimized the shrinkage parameter λ (like it was done in previous papers [7,40,41]). As for the *MetaboHealth*, we then used Spearman’s correlations to compare the different models and Cox proportional hazard models to investigate the associations with time to death.

## 3. Results

All comparisons are conducted on data gathered within the BBMRI.NL consortium (~35,000 samples in 28 cohorts, Methods Table 1). Samples were assayed using the Nightingale Health platform in multiple waves of data generation, as indicated with their respective years, 2014 and 2016. After the platform update by Nightingale of 2020, BBMRI.NL decided to re-quantify their dataset completely to have metabolomics features comparable to other consortia. It is important to stress that re-quantification consisted of a novel (computational) analyte quantification of the original assays performed in 2014 and 2016, i.e., no new samples were assayed. 

### 3.1. An Overview of Changes in Measured Metabolic Features 

With respect to marker availability, there are new and discontinued reported analytes. Notably, the latest version of the platform (2020 version) includes 37 analytes, which have been CE-approved for diagnostic purposes, i.e., ‘clinically validated’, making the Nightingale platform now not only interesting for epidemiological research, but also suited for use in the clinic [42]. In addition, 25 new analytes were added to the pool of metabolic markers now also readily measurable in EDTA plasma (Supplementary Materials). Moreover, the analyte pyruvate (pyr) is featured on the platform again, after being discontinued in 2016. Conversely, analytes showing insufficient replicability were discontinued, either already in the 2016 version (*dag*, *dagtg*, *fallen*, *cla*, *cla_fa*), or from 2020 onward (*hdl2_c* and *hdl3_c*), thus posing potential backward compatibility issues.

Looking more closely at the data, we also note some more subtle changes that nevertheless are helpful to highlight. Compared to older platform versions, the proportion of problematic values decreased in the re-quantified version of the platform, i.e., there are less values that failed to be detected (NaNs), were reported as zero, or were considered outliers (Figure S1). In addition, we observe that some markers were reported using different units between, and occasionally within, platform versions. For instance, albumin (alb) changed units from [signal area] in 2014 to [g/L] in 2020 (Figure S3). Particularly interesting are the different ranges of creatinine in the re-quantified measurements (2020 version), which in our case seems to depend on whether the first Nightingale metabolomics quantification was completed either in 2014 or in 2016, with reported units in mmol/L and μmol/L, respectively (Figure S2). These changes, if unnoticed, can impair replication of the results and application of multi-variate models.

### 3.2. Correlation Analyses of Metabolomics Measurements between Platform Versions

**Figure 1:**
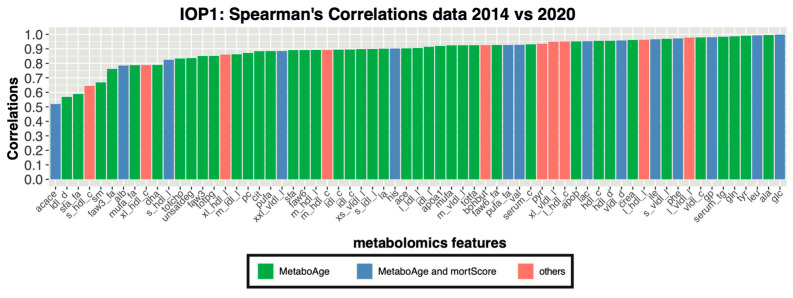
Evaluation of the metabolic markers before and after re-quantification in LLS-PAROFFS IOP1: Spearman’s correlations of the homonymous analytes measured in the first wave (2014) with their re-quantified version (2020), colored based on their use in *MetaboHealth* and *MetaboAge*.

First, we evaluated the Spearman’s correlation for each homonymous metabolic measurement across the different Nightingale platform versions within the Leiden Longevity study (LLS); a two-generation cohort containing highly aged individuals (LLS-SIBS) and their offspring with the relative partners (LLS-PAROFFS), with repeated measures over different time-points (IOP1, 2 and 3) (detailed description in Section 2). Considering same samples of LLS-PAROFFS IOP1, measured the first time in 2014 and re-quantified in 2020 (Figure 1), we observed that 36 out of the 65 homonymous non-derived analytes (55%), showed a Spearman’s correlation higher than 0.9, with one having a perfect value (*glucose*). Additionally, 24 had a medium Spearman’s correlation (0.7 ≤ ρ < 0.9), and only five analytes had a correlation lower than 0.7 (*acace, ldl_d*, *sfa_fa*, *s_hdl_c* and *sm*). Some analytes showed a shift in mean, presumably as a result of a recalibration step, as reflected by a change in levels, e.g., *ldl_d*: first wave [22.99 ÷ 25.5 nm] vs. re-quantified [23.4 ÷ 24.09 nm], or in units, e.g., *alb:* first wave [0.06 ÷ 0.14 signal area] vs. re-quantified [25.6 ÷ 62.78 g/l]. Furthermore, also 54 out of the remaining 169 analytes, mostly containing derived measures, showed lower Spearman’s correlations (R < 0.7) (Figure S4). 

**Figure 2:**
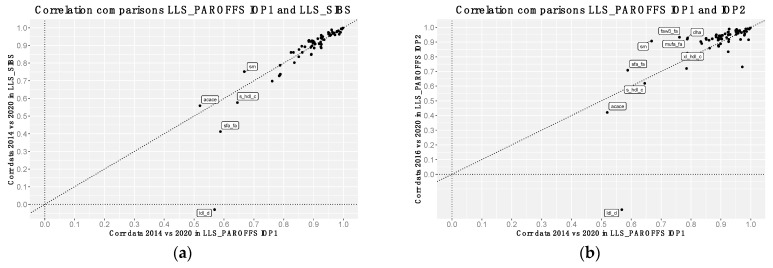
Comparisons of the Spearman’s correlations of the metabolites before and after re-quantification in different subgroups or platform versions: Each point of the scatterplots indicate the Spearman’s correlations of each metabolic markers before and after the re-quantifications in (**a**) LLS-PAROFFS IOP1 (*x* axis, first measured in 2014) and LLS-SIBS (*y* axis, first measured in 2014); and (**b**) LLS-PAROFFS IOP1 (*x* axis, quantification version 2014) with LLS-PAROFFS IOP2 (*y* axis, quantification version 2016). Metabolic markers were tagged if they show differences in Spearman’s correlations.

When computing the same correlation analyses comparing LLS_PAROFFS IOP1 (2014 data) with another cohort measured in the first wave, LLS-SIBS (2014 data), or with data of the same cohort of the second wave LLS_PAROFFS IOP2 (2016 data), we observe highly similar trends (Figure 2a and Figure S5A). While the majority of analytes show consistently high Spearman’s correlations with their re-quantified counterpart across waves and cohorts, we do observe some notable exceptions. Analytes with a low calibration Spearman’s correlation (ρ < 0.7) in the first data wave (either LLS_PAROFFS, or LLS-SIBS 2014 data) seem to show improvement in the second wave data (either LLS_PAROFFS, 2016 data), except for *ldl_d*. Considering that we find similar results also in LLS-PAROFFS IOP3 (Figure S5C,D), a second round of repeated measures quantified with the Nightingale platform 2016, we concluded that this latter platform version is more similar to the re-quantified data as compared to 2014 version. Similar results are maintained when enforcing the same samples sizes (Figure S6). 

**Figure 3:**
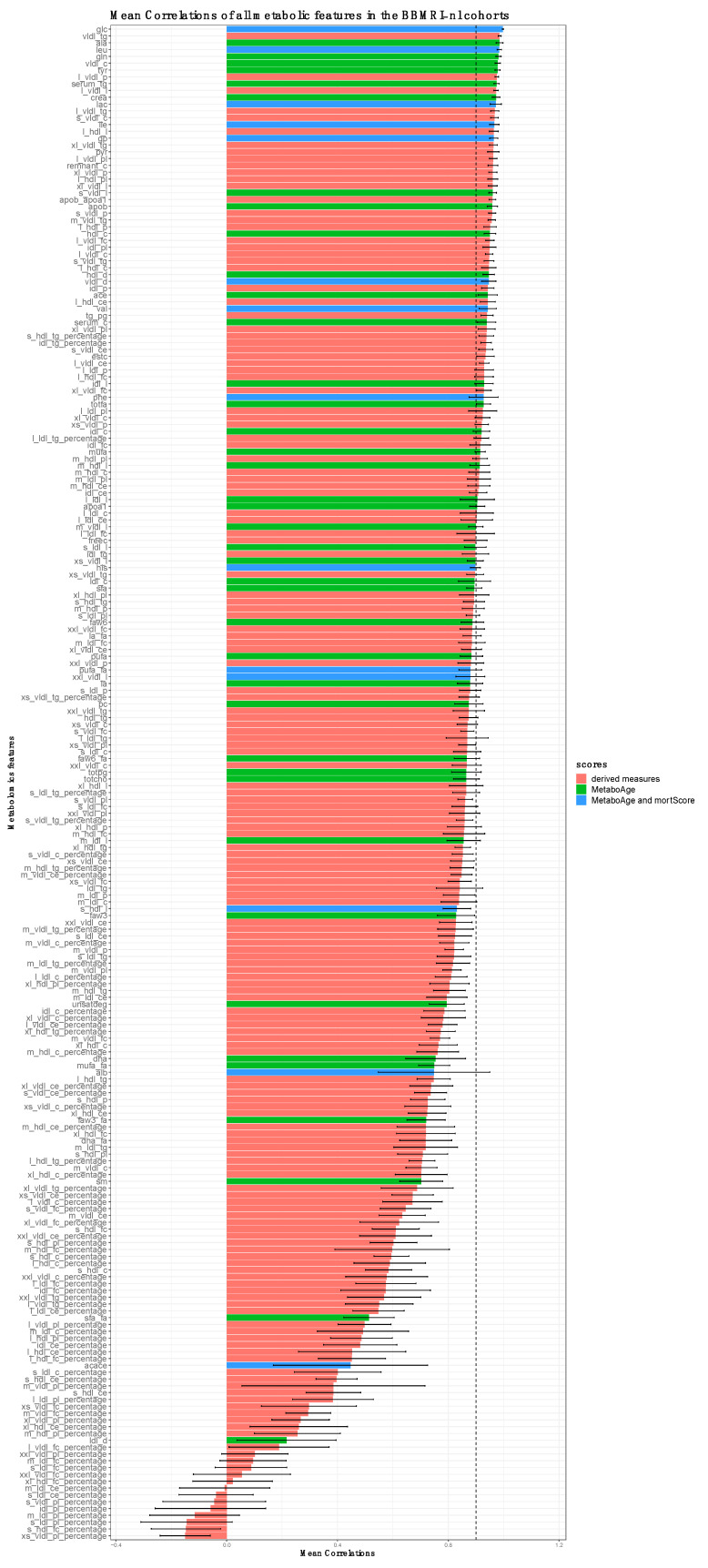
Spearman’s correlations of all the metabolomic analytes with itself in a different before (2014) and after re-quantification (2020) in all the BBMRI.NL cohorts. Each bar represents the mean and standard deviation of the Spearman’s correlation. The bars are colored based on their inclusion in *MetaboHealth* and *MetaboAge*. A vertical dotted line indicates a Spearman’s correlation of 0.9.

To investigate how the correlations of metabolomic features between the different Nightingale platform versions behave over different cohorts, we examined these on the whole BBMRI.NL dataset comprising 28 cohorts (Figure 3). Observed Spearman’s correlations vary between −0.5 (generally for derived analytes, such as ratios or percentages) and perfect positive correlation (*glucose*). The lower correlations were not due to a lower variance in the markers (Figure S7D). Even though there are some cohorts that show generally lower Spearman’s correlations for all the analytes (e.g., BIOMARCS, or STEMI-GIPS), the other cohorts show consistent correlations for the different analytes (Figure S7B,C). 73 analytes had a mean Spearman’s correlation above 0.9 across all BBMRI.NL biobanks (Figure 3 and Figure S7A, Table S5). 27 (48%) and 8 (57%) of these analytes overlap with the 57 and 14 analytes that were used to construct the *MetaboAge* and *MetaboHealth* score, respectively (Figure S7E).

### 3.3. The Clinically Validated Biomarkers Show Similar Correlation, but Improved Calibration with Respect to Previous Quantification

**Figure 4:**
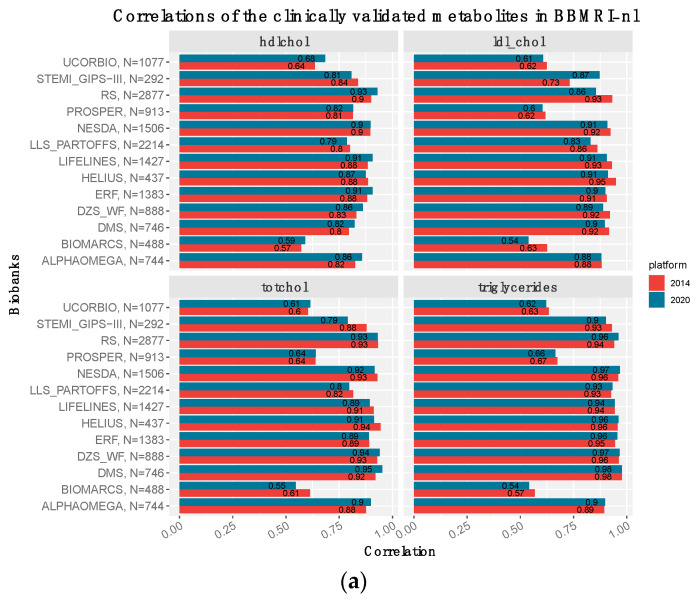

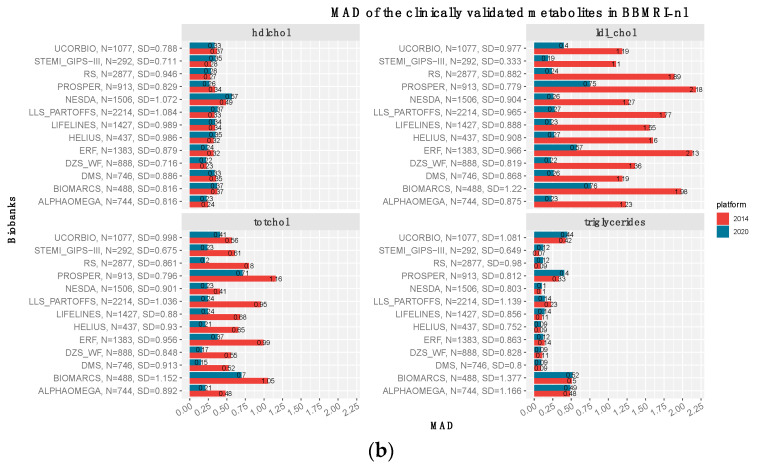
Comparisons of Nightingale metabolomics markers, measured in 2014 (red) and 2020 (blue), with the clinically measured values in BBMRI.NL: Bar-plots of the (**a**) Spearman’s correlations and (**b**) the median absolute distance (MAD) of the *hdl cholesterol, ldl cholesterol* (calculated with the Friedewald equation), *total cholesterol* and *triglycerides* calculated with clinical chemistry, with their corresponding values in the Nightingale assay (*hdl_c, ldl_c/clinical_ldl_c, serum_c* and *serum_tg*). The label on the *y*-axis indicates the biobank, the total number of samples with available quantification and the standard deviation of the clinically measured metabolite.

The latest Nightingale metabolomics platform contains 37 analytes approved by the European community for diagnostics [42]. This is particularly interesting for Consortia like BBMRI.NL, as it allows for an efficient quantification of various routinely assessed clinical biomarkers in one single platform. For this purpose, we evaluated to what extent previously measured clinical variables within BBMRI.NL align with their corresponding analytes on the Nightingale platform. Four of the thirty-seven clinical biomarkers (*HDL-cholesterol, LDL-cholesterol, triglycerides*, and *total cholesterol*) were available in thirteen of the twenty-eighty cohorts (14,995 samples, Figure 4) and showed a medium to high Spearman’s correlation in most of the cohorts, apart for BIOMARCS, PROSPER, and UCORBIO [mean ρ = 0.6]. While different Nightingale versions generally showed very similar correlations with their clinical chemistry counterparts, notable differences are observed when considering the median absolute distance (MAD). For the 2020 version, we observe an improved concordance between clinically measured biomarkers and their Nightingale counterpart, particularly for *LDL-cholesterol* and *total cholesterol*. Evaluation of additional clinical variables (*glucose, creatinine, and albumin*) within our in-house cohort LLS, indicated that this observation of similar Spearman’s correlations, accompanied by an improved MAD for platform version 2020 can be extended to other analytes that have been clinically validated (Figure S9).

### 3.4. The MetaboHealth Score Shows a Comparable Association with Mortality Using Re-Quantified Data

**Figure 5:**
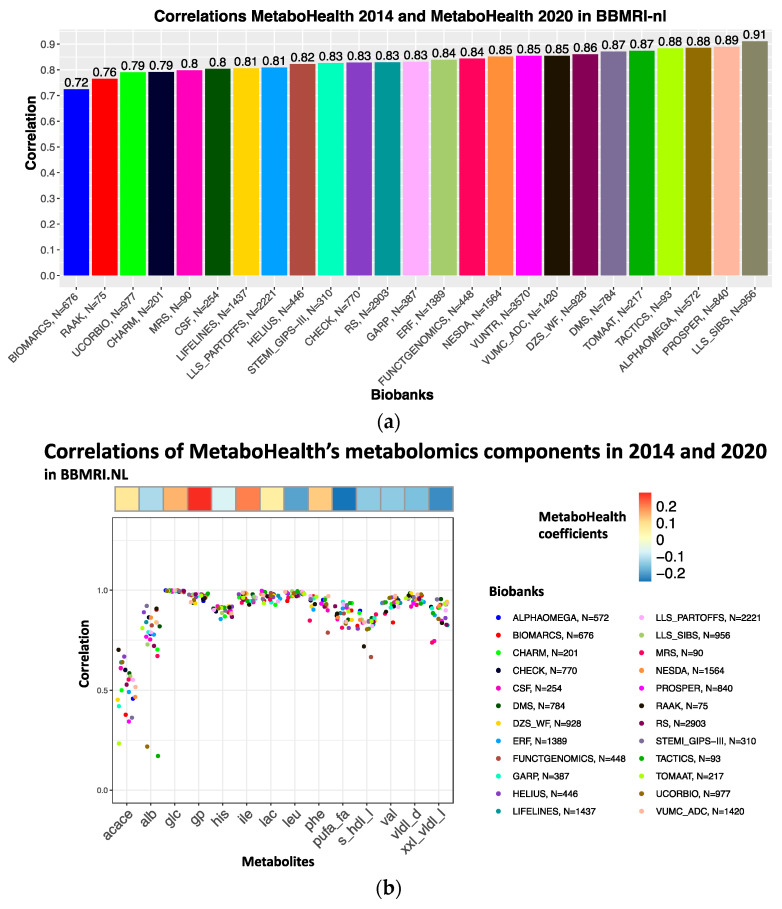
*MetaboHealth* score consistency over BBMRI.NL: (**a**) Bar-plot presenting the Spearman’s correlation of the *MetaboHealth* score calculated in all the BBMRI.NL biobanks with the metabolites in the data measured in 2014 or 2020; (**b**) Jitter-plot of the Spearman’s correlations of the metabolic markers used to build the *MetaboHealth* score calculated in data 2014 and 2020, divided per biobank. The heatmap on top shows the coefficients of each biomarker in the *MetaboHealth* score.

Next, we evaluated whether the platform changes affected the replication of the *MetaboHealth* score [2]. The *MetaboHealth* score correlated on average ρ~0.83 between the 2014 platform and the re-quantification in 2020 over all the cohorts (Figure 5a); with a maximum of ρ=0.91 (in LLS-SIBS) and a minimum of ρ=0.72 BIOMARCS. Higher Spearman’s correlations for LLS-SIBS [89÷103 y.o.] and PROSPER [70÷85 y.o.] might be explained by the stronger signal caused by the fact that these cohorts generally include older individuals, with a high frequency of mortality or cardiovascular events. Cohort-specific differences in correlations between platform versions could be explained by inconsistent correlations of *acace*, *albumin*, *s_hdl_l,* and *xxl_vldl_d* that have relatively high coefficients in the *MetaboHealth* score (in Figure 5b). Indeed, we notice that patient cohorts such as BIOMARCS, RAAK, and UCORBIO do have lower Spearman’s correlations. 

**Figure 6:**
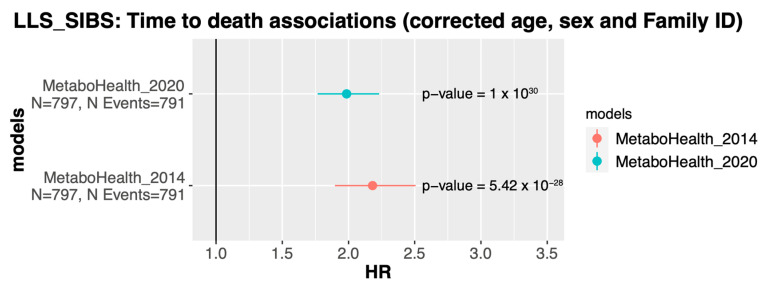
*MetaboHealth* score associations with time-to-death in LLS-SIBS: Association with time-to-death of the *MetaboHealth* score calculated with the metabolic markers quantified in 2014 (*MetaboHealth_2014*) and the metabolic markers quantified in 2020 (*MetaboHealth_2020*). The two Cox regression models were performed on 797 individuals with 791 reported deaths and corrected for age, sex, and Family relationships.

Since the *MetaboHealth* score maintained similar predictions in the platform with re-quantified metabolites, we next were interested whether the re-quantified score also showed similar associations with mortality. To this end, we modeled time-to-death using a Cox proportional Hazards model, while adjusting for age, sex, and family relation, in LLS-SIBS (N_total_ = 797, N_events_ = 791). Both versions remained significantly associated (2014: HR~2.18, *p* = 5.42 × $10^{-28}$, and 2020: HR~1.98, *p* = 1 × $10^{-30}$) albeit with a slightly attenuated effect size for the 2020 platform version (Figure 6).

### 3.5. A Retrained MetaboAge on Re-Quantified Data Shows Similar Associations with Mortality Compared to the Previous Version of MetaboAge

**Figure 7:**
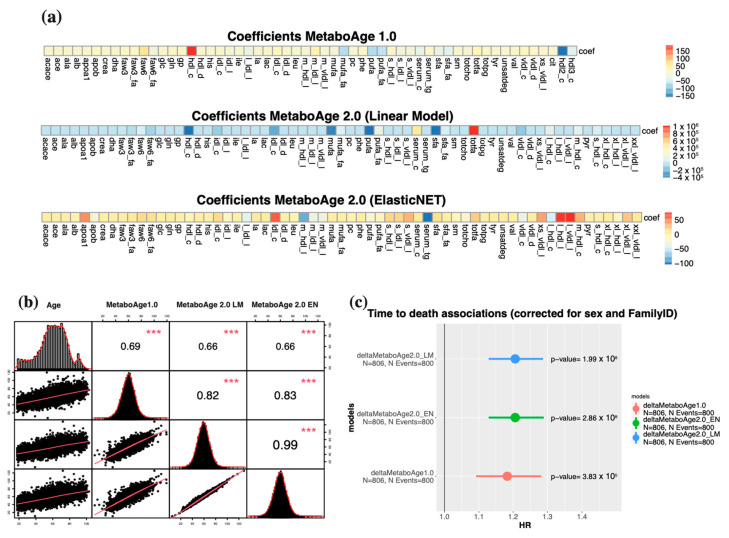
*MetaboAge 2.0* evaluations: (**a**) Coefficients of *MetaboAge*1.0 and *Metaboage*2.0 ordered in the same manner; (**b**) Spearman’s correlation between age, *MetaboAge*1.0, *MetaboAge*2.0 linear model (LM) and ElasticNET (EN). *** indicate significant correlation (*p* value < 0.05); (**c**) Associations of time-to-death with the three age predictors.

Since two essential variables (*hld2_c* and *hdl3_c*) were discontinued in the 2020 platform, the original *MetaboAge* model (*MetaboAge 1.0*) could not be computed [1]. Therefore, we decided to retrain the *MetaboAge* model using the re-quantified Nightingale 2020 measurements, either using a: (1) a linear model (LM), consistent with the previous *MetaboAge* model; and (2) an elastic net regression (EN), regularizing the contributions of each individual metabolite. 5-Fold Cross Validation, over the BBMRI.NL dataset (~20,366 samples, after quality control), showed overall similar accuracies, with a slight advantage for the linear model (*MetaboAge 2.0*: LM, ρ2 = 0.451; EN, ρ2 = 0.449, Figure S10). Spearman’s correlations between the old and new versions of the models over all the BBMRI.NL biobanks showed cohort-specific differences, with low correlations in the RAAK cohort (ρ = 0.5) and moderately to high correlations for the ERF and FUNCTGENOMICS cohorts (ρ = 0.85 and 0.86, respectively) (Figure S11). Nonetheless, we observe an overall high correlation between the two novel versions of the MetaboAge models (ρ = 0.99) (Figure 7B), despite that the informative metabolomics features are quite different across the three models (Figure 7A). Yet, the elastic net version has a slightly higher Spearman’s correlation with the *MetaboAge 1.0* (LM: R = 0.82 and EN: ρ = 0.83, Figure 7A). Nonetheless, the linear model assigns higher coefficients to only few features compared to the elastic net model (Figure 7A) (*MetaboAge 1.0* [range: −150,150], *MetaboAge 2.0*: LM [range: −40,000, 1,000,000], *MetaboAge 2.0*: EN [range: −100, 50]). 

Finally, we performed a Cox-regressions analysis to predict time-to-death (corrected for age sex and family relation) in the LLS-SIBS cohort (Ntotal = 806, Nevents = 800) (Figure 7C). The associations with mortality are quite similar (equivalently significant and moderate effect sizes) across all models, but slightly higher for the *MetaboAge 2.0* models (LM: HR~1.2, *p* = 1.69 × $10^{-8}$ and EN: HR~1.2, *p* = 2.39 × $10^{-8}$, *MetaboAge 1.0*: HR~1.18, *p* = 2.89 × $10^{-5}$).

## 4. Discussion

Using the BBMRI.NL biobanking consortium, we evaluated the replicability across Nightingale Health platform updates between 2014, 2016, and 2020 (re-quantification). We observe improvements regarding the overall quantification quality; i.e., a decrease in missingness; lower numbers of values that are reported as zero; and a better concordance with clinical measurements. On the other hand, there are discontinued metabolites, and changes in reported units between and sometimes within quantification versions that could affect replication efforts. Some analytes displayed low calibration Spearman’s correlations between the 2014/2016 and 2020 platform versions. Moreover, the 2016 version resulted to be more similar to the re-quantified data as compared to the 2014 version, even when evaluating the same sample sizes. Replication over the BBMRI-nl cohorts indicated similar results, however, with lower concordance for some studies (e.g., BIOMARCS, or STEMI-GIPS). Nevertheless, our analyses revealed a list of 73 analytes being highly consistent between quantification versions of the BBMRI.NL data set (mean R > 0.9). Additionally, the re-quantification demonstrated its effectiveness in a substantial reduction of the median absolute distance (MAD) in our comparisons with four clinically assessed lipid-related features over thirteen cohorts. Moreover, the *MetaboHealth* score did generally replicate well between platform version in the BBMRI-nl cohorts (mean ρ = 0.83, min ρ = 0.72, BIOMARCS, and max ρ = 0.91, LLS-SIBS). Lower Spearman’s correlations were attributed to inconsistencies in some score-related analytes (*acace*, *albumin*, *s_hdl_l* and *xxl_vldl_d*). Importantly, the time-to-death association of the *MetaboHealth* score was not significantly affected by the platform updates [2]. We retrained the MetaboAge score in BBMRI-nl due to the absence of two analytes in the new platform version [1]. Spearman’s correlations with the original MetaboAge model (*MetaboAge 1.0*) showed moderately high concordance over all cohorts in BBMRI.NL, apart for RAAK (ρ~0.5), which is a relatively small cohort focusing on patient with osteoarthritis. Notably, the retrained version of MetaboAge did recapitulate the previously reported association with time-to-death. Between the two versions of the *MetaboAge 2.0*, we believe the elastic net version to be the better model as the regularization should warrant a higher robustness to future changes of the platform [43].

A significant constraint of the present study is that we do not have access to the details of the changes performed to the quantification algorithms, as this is proprietary information of the company, hence we limit ourselves to analyzing the differences in their output and their consequences. Another major limitation, concerns the assessment of the recent platform re-quantification of the Nightingale Health Plc, is the limited availability of clinically measured features for comparisons. While the algorithm updates primarily sought European approval for thirty-seven analytes [42], our evaluations were restricted to just four lipid related metrics. Despite this limitation, in view of the platform’s strong lipid focus, we believe that the comparisons at our disposal continue to hold substantial value.

In conclusion, replication of previous findings and analysis of repeated measures is one of the cornerstones of epidemiological research [44,45]. Hence, we call for caution when utilizing Nightingale data quantified at different time points. Moreover, it is important to realize that pre-trained metabolic models cannot readily be applied across different versions of the data. In these circumstances, we recommend a retraining of the score, or, if this is not possible, an extensive re-evaluation of the models and their associations with endpoints.

## Figures and Tables

**Table 1:** Data and platform versions available in BBMRI-NL.

Waves	N. Samples	N. Biobanks	Platform Version
First wave	24,994	26	Version 2014
Second wave	9880	10	Version 2016
Re-quantifications	34,015	28	Version 2020

N = number.

**Table 2:** Data and platform versions available in the Leiden Longevity study.

**LLS-PAROFFS [30–79 years old]**						
	Wave	Platform version first measure	Re-quantification	Total N. samples	N. samples after QC	Drop rate (%)
IOP1	First Wave	Version 2014	Version 2020	2313	1925	16.77
IOP2	Second Wave	Version 2016	Version 2020	670	604	9.85
IOP3	Third Wave	Version 2016	Version 2020	498	400	19.68
**LLS-SIBS [89–103 years old]**						
IOP1	First Wave	Version 2014	Version 2020	998	948	5.01

N = number.

## Data Availability

The data are available upon request at https://www.bbmri.nl/, last access 1 October 2023. A presentation of the results with the code to reproduce this work can be found at (https://github.com/DanieleBizzarri/NightingaleMetabolomics_Requantification2020, last access 18 November 2023).

## Supplementary Materials

The following supporting information can be downloaded at: https://www.mdpi.com/article/10.3390/metabo13121181/s1, Figure S1:Missingness in the platform; Figure S2: Distribution of creatinine in the Nightingale Metabolomics dataset of 2020; Figure S3: Comparison albumin units; Figure S4: Spearman’s correlations comparing the 2014 and 2020 versions; Figure S5: Comparisons of the correlations of the metabolites before and after re-quantification in LLS-SIBS and LLS-PAROFFS IOP1, IOP2, and IOP3; Figure S6: Comparisons of correlations with the same sample numbers; Figure S7: Spearman’s correlations of all 220 metabolomics analytes in all the biobanks of BBMRI-nl before (2014) and after the re-quantifications (2020); Figure S8: Medians of the Spearman’s correlations; Figure S9: Comparisons with the clinically measured values in LLS; Figure S10: Correlations MetaboAge 2.0 with age; Figure S11: Correlations MetaboAge 2.0 with MetaboAge 1.0; Table S1: Metabolites utilized to train MetaboAge; Table S2: Discontinued metabolites; Table S3: Metabolites added with Nightingale platform version 2020; Table S4: Clinically validated metabolites; Table S5: stable metabolites. Consortium Banner. Cohort Description. Acknowledgements. Ethics Statements.
